# Supplementary figures and images for: α-Synuclein fibrils subvert lysosome structure and function for the propagation of protein misfolding between cells through tunneling nanotubes
Source: PLoS Biol. 2021 Jul 20;19(7):e3001287. doi: 10.1371/journal.pbio.3001287 (PMC8291706; doi:10.1371/journal.pbio.3001287)

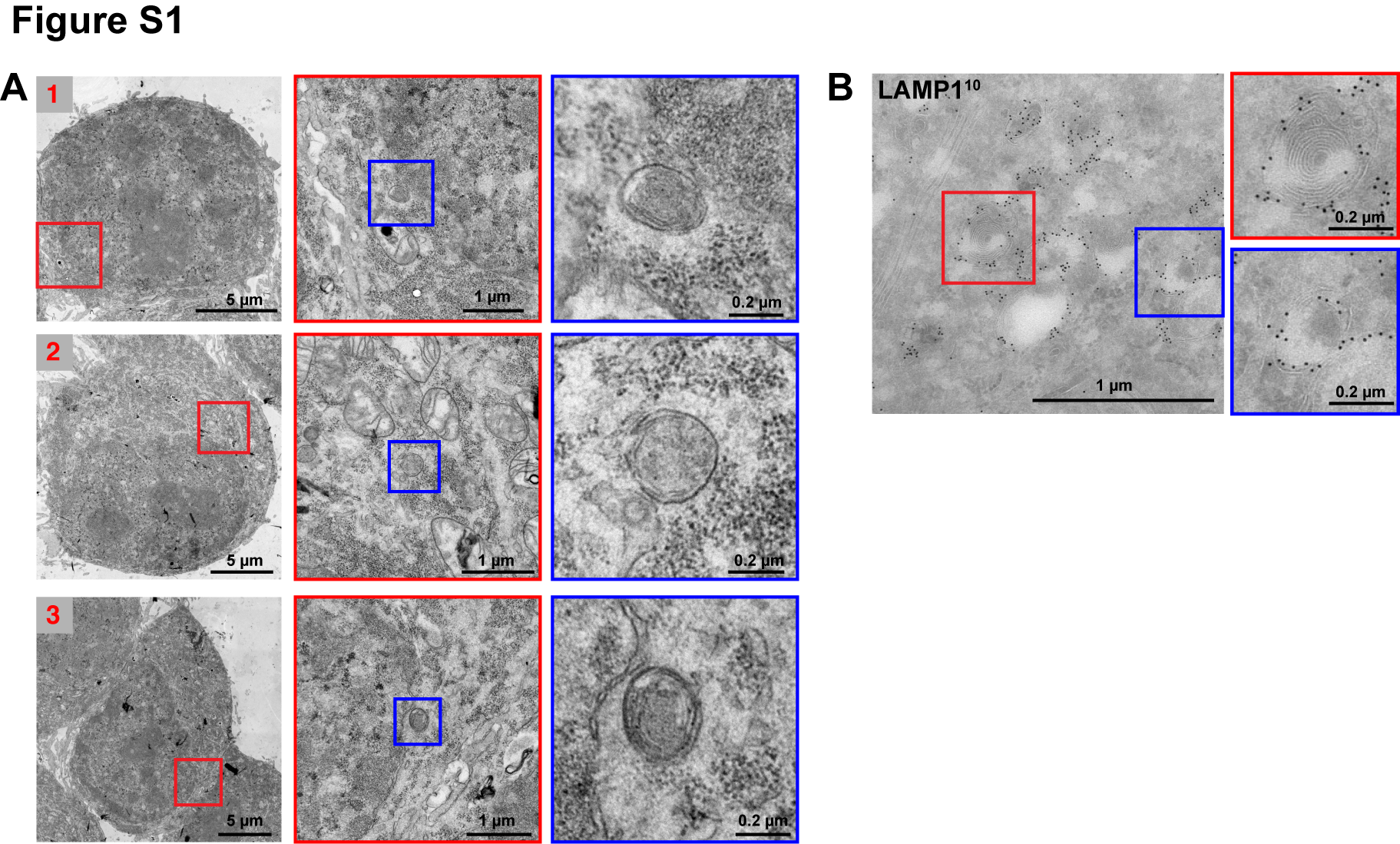

Supplement: S1 Fig — (A) Resin-embedded EM images of control CAD cells. Three examples of control lysosomes (indicated by blue squares) selected from different regions (indicated by red squares) of 3 different cells are presented. Scale bar for the cells: 5 μm, for the selected regions: 1 μm, and for lysosomes: 0.2 μm. (B) On-section EM images of control CAD cells immunogold labeled with LAMP110. Two examples of lysosomes were presented in insets selected from regions indicated by red and blue squares. Scale bar: 1 μm (for the insets: 0.2 μm). CAD, Cath.a-differentiated; EM, electron microscopy; LAMP, lysosome-associated membrane protein. (TIF) [file pbio.3001287.s001.tif]

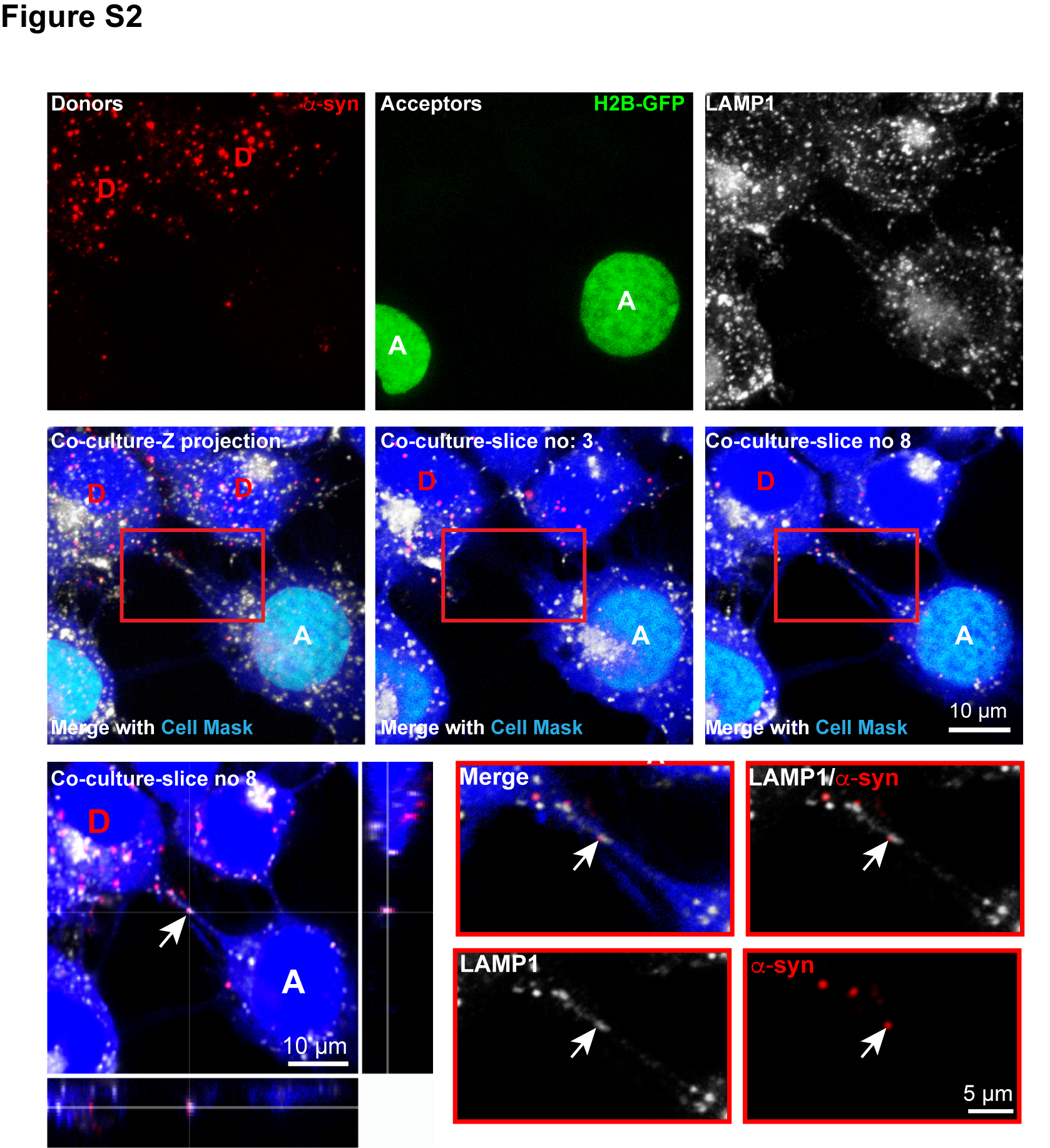

Supplement: S2 Fig — Representative image of a TNT having a lysosome containing α-syn fibrils which is formed between α-syn loaded donor cell for 18 hours (D) and H2B-GFP transfected acceptor cell (A) in 24 hours of CAD-CAD coculture. Z projection of donor and acceptor cells and lysosomes labeled with LAMP1 is presented (upper panels). Z projection of merged image with an additional staining of HCS CellMask Blue, a bottom section (section no: 3) where TNT is not visible and an upper section (section no: 8) where TNT is visible are presented (middle panels). Arrows in the orthogonal view of the upper section and in insets are indicating the lysosome containing α-syn fibrils inside of the TNT (lower panels). Scale bar: 10 μm (for insets: 5 μm). α-syn, α-synuclein; CAD, Cath.a-differentiated; LAMP, lysosome-associated membrane protein; TNT, tunneling nanotube. (TIF) [file pbio.3001287.s002.tif]

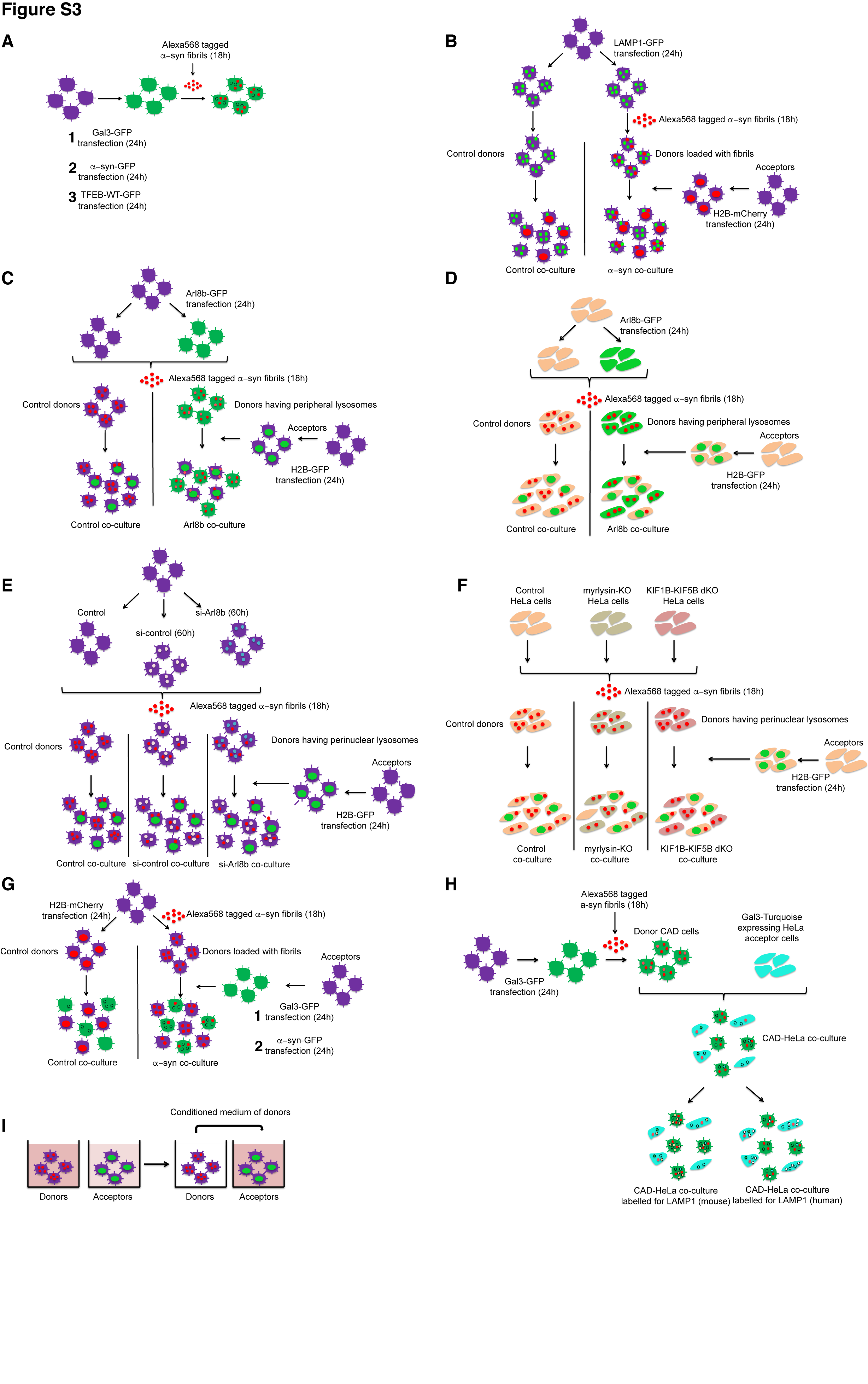

Supplement: S3 Fig — (A) CAD cells were transiently transfected with Gal3-GFP (1), α-syn-GFP (2), or TFEB-WT-GFP (3) and treated with Alexa 568–tagged α-syn fibrils for 18 hours; cells were then analyzed for the presence of Gal3-GFP puncta, α-syn-GFP puncta, or nuclear TFEB, respectively. (B) Donor CAD cells were transiently transfected with LAMP1-GFP and either treated with Alexa 568–tagged α-syn fibrils for 18 hours (coculture prepared from these donors was referred to as “α-syn coculture”) or left untreated (coculture prepared from these donors was referred to as “control coculture”). Donor cells were then cocultured with acceptor CAD cells transiently transfected with H2B-mCherry for 24 hours. Efficiency of the LAMP1-GFP+ lysosome transfer was measured in each condition. (C) Donor CAD cells were transiently transfected with Arl8b-GFP (coculture prepared from these donors was referred to as “Arl8b coculture”) or not (coculture prepared from these donors was referred to as “control coculture”) prior to be loaded with Alexa 568–tagged α-syn fibrils for 18 hours. Arl8b-GFP expressing donors (having more peripheral lysosomes) and control donors were then cocultured with acceptor CAD cells that were transiently transfected with H2B-GFP for 24 hours. Efficiency of the α-syn fibrils’ transfer was measured in each condition. (D) Donor HeLa cells were transiently transfected with Arl8b-GFP (coculture prepared from these donors was referred to as “Arl8b coculture”) or not transfected (coculture prepared from these donors was referred to as “control coculture”) prior to be loaded with Alexa 568–tagged α-syn fibrils for 18 hours. Arl8b-GFP expressing donors (having more peripheral lysosomes) and control donors were then cocultured with acceptor HeLa cells that were transiently transfected with H2B-GFP for 24 hours. Efficiency of the α-syn fibrils’ transfer was measured in each condition. (E) Three donor CAD cell populations were prepared: untreated control cells, cells pretreated with sicontrol [file pbio.3001287.s003.tif]

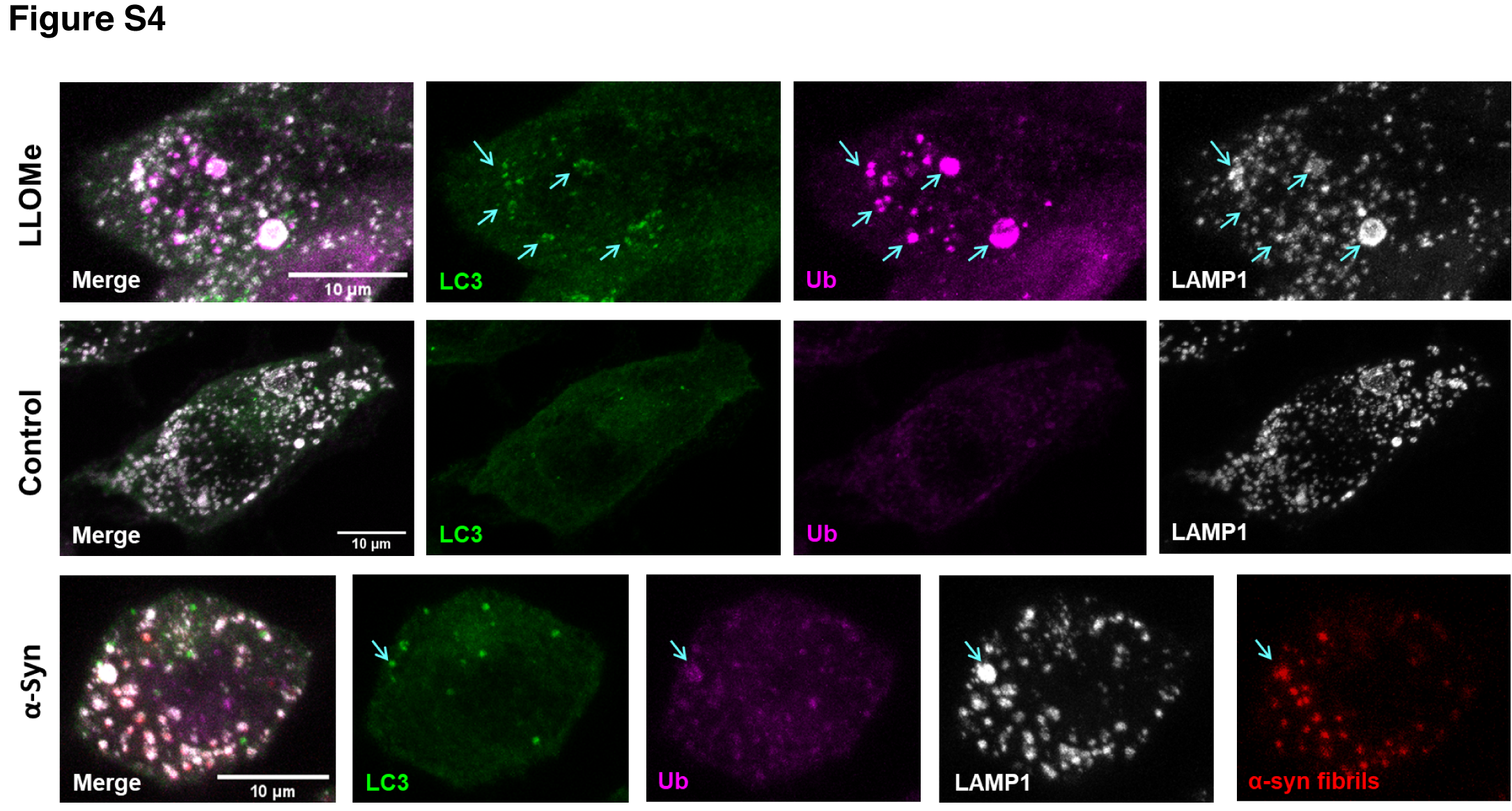

Supplement: S4 Fig — Representative confocal images of CAD cells control, treated with 1 mM LLOMe for 3 hours or treated with Alexa 568–tagged α-syn fibrils for 18 hours and immunolabeled with LC3-Alexa 488, Ubiquitin FK2-Alexa 647, and LAMP1- CF405M (pseudo colored in gray) antibodies. Light blue arrows indicate lysosomes under lysophagy. n = 3 (60 cells analyzed per condition). Scale bar: 10 μm. α-syn, α-synuclein; CAD, Cath.a-differentiated; LAMP, lysosome-associated membrane protein; LC3, Microtubule-associated protein 1A:1B-light chain 3; Ub, Ubiquitin; LLOMe, L-leucyl-L-leucine methyl ester. (TIF) [file pbio.3001287.s004.tif]

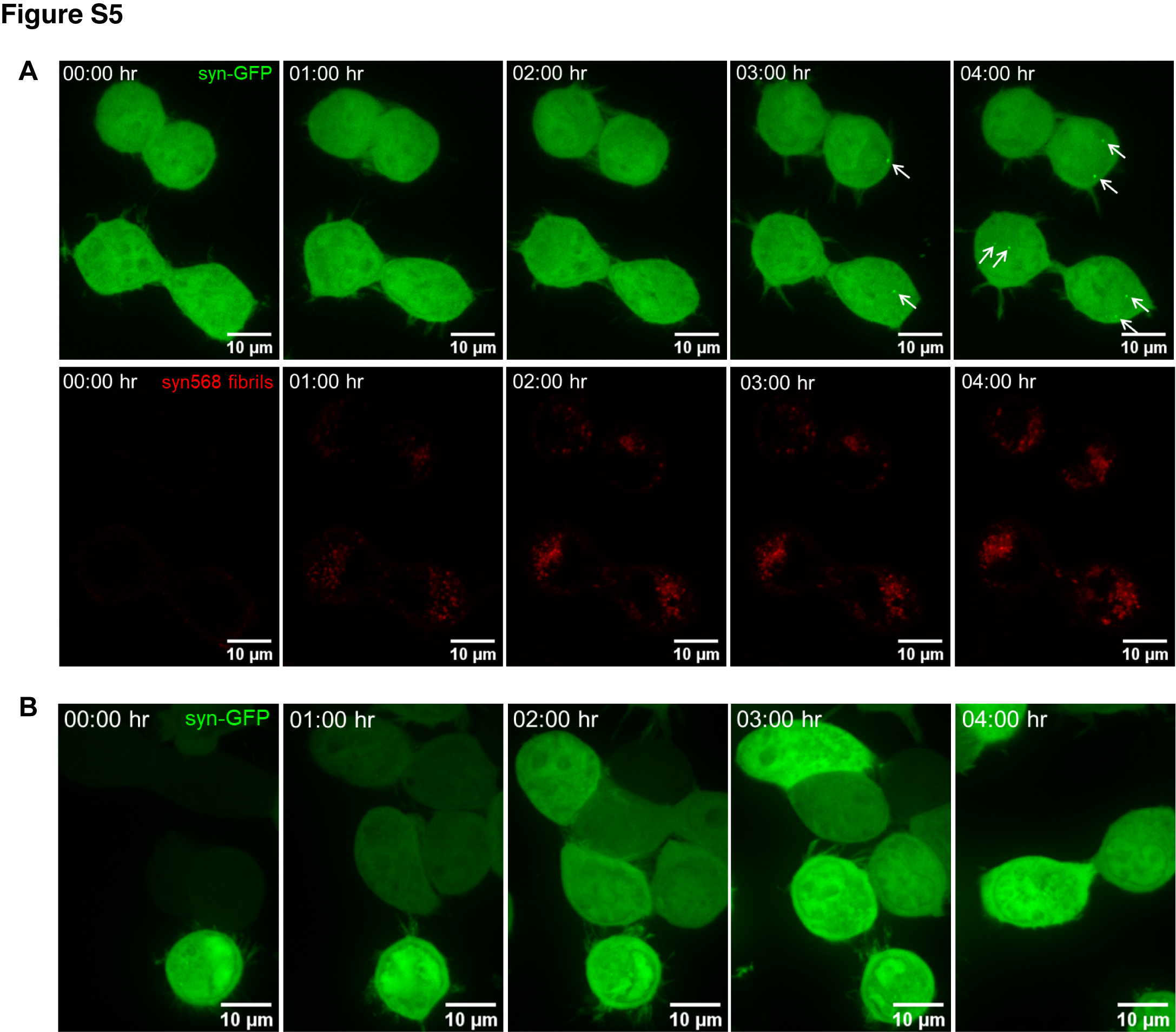

Supplement: S5 Fig — (A) Time frames at 0/1/2/3/4 hours of the S1 Movie of CAD cells overexpressing soluble α-syn-GFP monitored after the administration of α-syn fibrils. (B) Time frames at 0/1/2/3/4 hours of the S2 Movie of CAD cells overexpressing soluble α-syn-GFP. α-syn, α-synuclein; CAD, Cath.a-differentiated. (TIF) [file pbio.3001287.s005.tif]

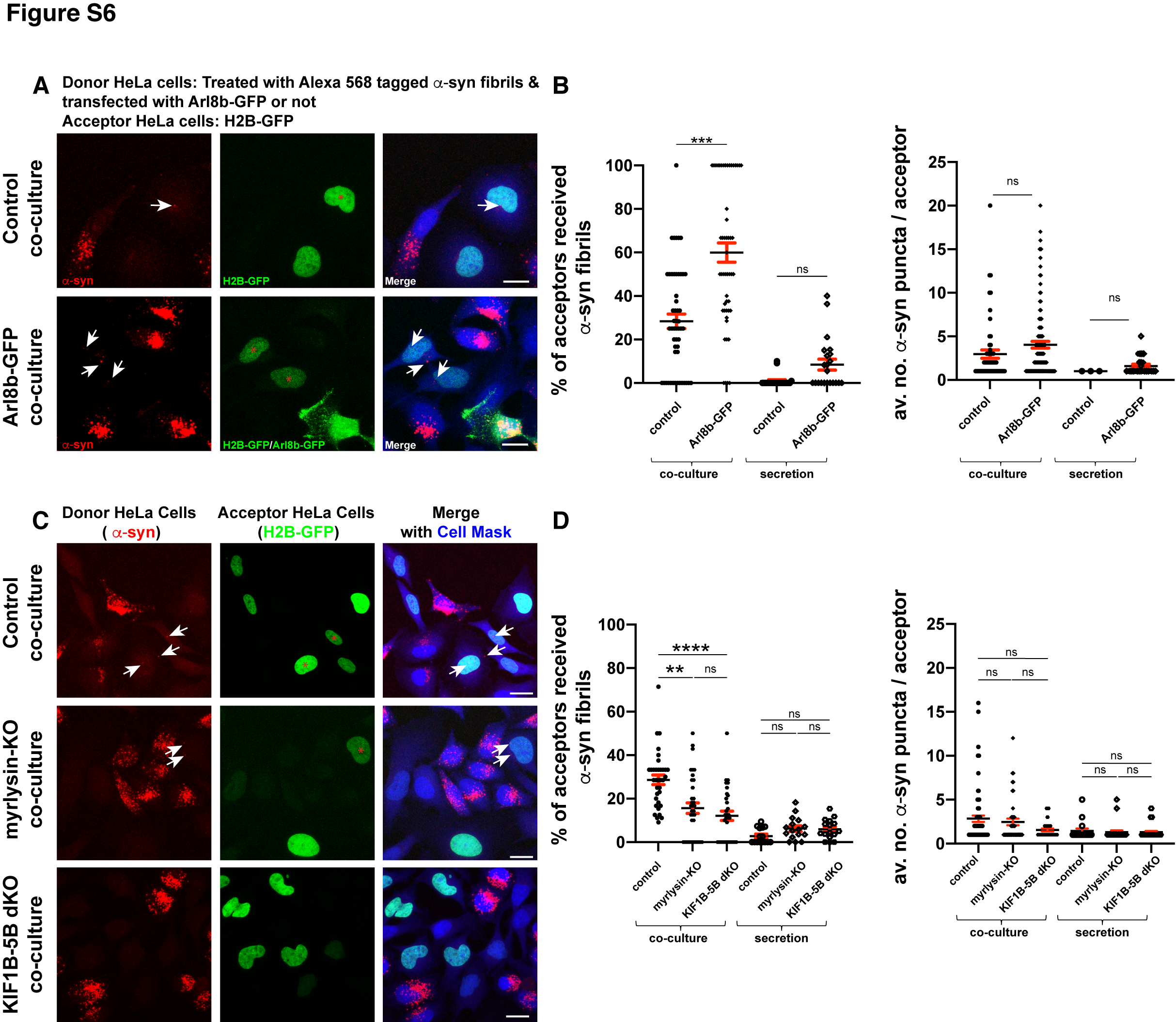

Supplement: S6 Fig — (A) Representative confocal images of donor and acceptor HeLa cells after 24 hours of coculture. Donor cells were either treated with Alexa 568–tagged α-syn fibrils for 18 hours (referred as “control coculture”) or were transfected with Arl8b-GFP prior to be treated with α-syn fibrils (referred as “Arl8b-GFP coculture”). Acceptor cells were transfected with H2B-GFP. Moreover, 24 hours later, cocultures were labeled with HCS CellMask Blue and DAPI. Arrows indicate the α-syn fibrils received by acceptor cells, and asterisks indicate the cells having α-syn fibrils in each coculture condition. Scale bar: 10 μm. (B) % of acceptor cells received α-syn fibrils in control coculture (28 ± 3%), Arl8b-GFP coculture (60 ± 5%), control secretion (1 ± 1%), and Arl8b-GFP secretion (8 ± 3%) is presented (left graph); average number of α-syn puncta in control coculture (3 ± 1), Arl8b-GFP coculture (5 ± 1), control secretion (1 ± 0.0), and in Arl8b-GFP secretion (2 ± 0.2) is presented (right graph). Mean ± SEM, n = 3 (70 acceptor cells per condition). ns = not significant, ***P = 0.0001 by Kruskal–Wallis nonparametric ANOVA test followed by Dunn multiple comparison tests. (C) Representative confocal images of donor and acceptor HeLa cells after 24 hours of coculture. WT, myrlysin-KO, and KIF1B-5B dKO HeLa cells were loaded with Alexa 568–tagged α-syn fibrils (referred as control, myrlysin-KO, and KIF1B-5B dKO cocultures, respectively) and cocultured with acceptor cells transfected with H2B-GFP. Cocultures were labeled with HCS CellMask Blue and DAPI. Arrows indicate the α-syn fibrils received by acceptor cells, and asterisks indicate the cells having α-syn fibrils in each coculture condition. Scale bar: 10 μm. (D) % of acceptor cells received α-syn fibrils in control coculture (29 ± 2%), myrlysin-KO coculture (16 ± 2%), KIF1B-5B dKO coculture (12 ± 2%), control secretion (5 ± 2%), myrlysin-KO secretion (6 ± 1%), and KIF1B-5B dKO secretion (6 ± 1%) is presented (left graph); average n [file pbio.3001287.s006.tif]

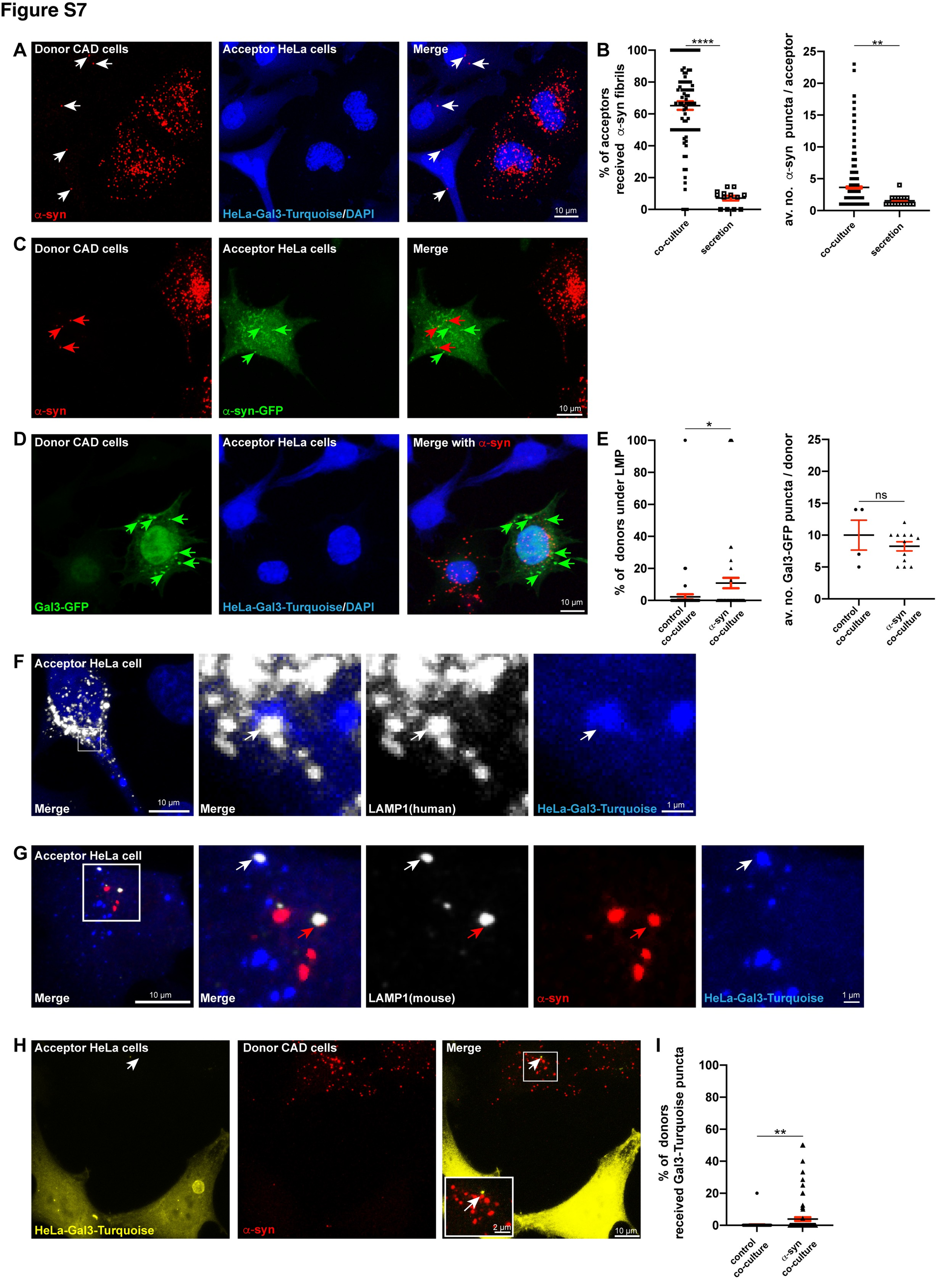

Supplement: S7 Fig — (A) Representative confocal image of donor CAD cells loaded with Alexa 568–tagged α-syn fibrils (18 hours) cocultured with HeLa Gal3-Turquoise acceptor cells for 24 hours. In acceptor HeLa cells, arrows indicate α-syn fibrils. Scale bar: 10 μm. (B) % of acceptor HeLa cells received α-syn fibrils in coculture (65 ± 3%) and in secretion test (7 ± 2%) is presented (left graph). Mean ± SEM. ****P < 0.0001 by 2-tailed t test. Average number of α-syn fibrils puncta per acceptor cell in coculture (4 ± 0.2) and in secretion test (2 ± 0.2) is presented (right graph). ns = not significant by Mann–Whitney U test. (C) Representative confocal image of donor CAD cells loaded with Alexa 568–tagged α-syn fibrils (18 hours) cocultured with acceptor WT HeLa cells transfected with α-syn-GFP for 24 hours. Red arrows indicate α-syn fibrils, and green arrows indicate α-syn-GFP puncta formation (seeding) in acceptor HeLa cells. Scale bar: 10 μm. (D) Representative confocal image of Gal3-GFP transfected and Alexa 568–tagged α-syn fibril loaded donor CAD cells (18 hours) cocultured with HeLa Gal3-Turquoise cells for 24 hours. In donor CAD cell, green arrows indicate Gal3-GFP puncta formation. Scale bar: 10 μm. (E) % of donor CAD cells under LMP in control (2 ± 2%) and in α-syn (11 ± 4%) cocultures is presented (left graph); average number of Gal3-GFP puncta in donor cells under LMP in control (10 ± 2) and in α-syn (14 ± 4) cocultures is presented (right graph). Mean ± SEM. ns = not significant, by *P = 0.04 Mann–Whitney U test. (F) HeLa Gal3-Turquoise acceptor cell under LMP labeled for LAMP1 human Alexa 647 antibody (pseudo colored in gray). Colocalization between Gal3-Turquoise puncta and LAMP1 (indicated by arrows) is presented in a selected region indicated by the square. Scale bar: 10 μm (for the inset: 1 μm). (G) HeLa Gal3-Turquoise acceptor cell under LMP labeled with LAMP1 mouse Alexa 647 antibody (pseudo colored in gray). Colocalization between Gal3-Turquoise puncta and donor’s lys [file pbio.3001287.s007.tif]
