# Supplementary material for: α-Synuclein fibrils subvert lysosome structure and function for the propagation of protein misfolding between cells through tunneling nanotubes
Source: PLoS Biol. 2021 Jul 20;19(7):e3001287. doi: 10.1371/journal.pbio.3001287 (PMC8291706; doi:10.1371/journal.pbio.3001287)
Supplement: S1 Raw Images — Raw western blot images related to Figs 2A, 6C and 7C are presented. (PDF) [file pbio.3001287.s011.pdf]

WB Lamp1 – Fig 2A

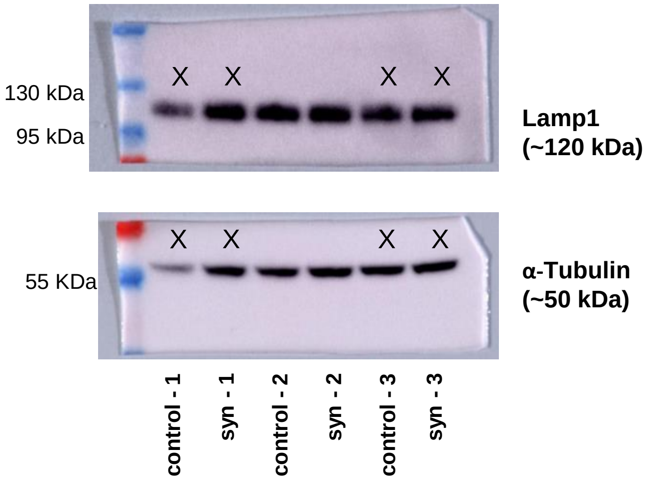

Cell lysates (30 µg/lane) of CAD cells treated for 18 h with α-synuclein fibrils (syn) or not (control).

The specific protein bands were visualized using the ECL-immunoblotting chemiluminescence system (GE Healthcare Life sciences) and the ImageQuant LAS 500TM camera (GE Healthcare Life sciences).

From this original image we generated the image in the Fig 2A.

WB TFEB – Fig 6C

(1st incubation with rabbit anti-TFEB antibody) (2nd incubation with rabbit anti-GAPDH antibody)

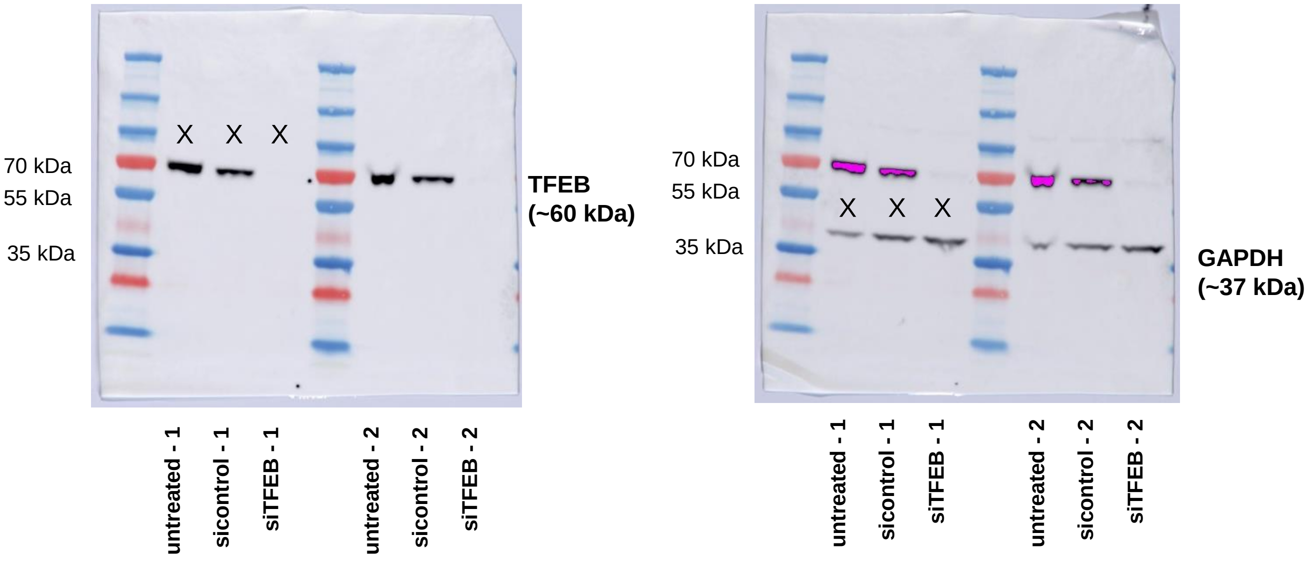

Cell lysates (30 µg/lane) of CAD cells untreated (untreated) or treated for 60 h with siRNA scramble (sicontrol)/siRNA TFEB (siTFEB).

The specific protein bands were visualized using the ECL-immunoblotting chemiluminescence system (GE Healthcare Life sciences) and the ImageQuant LAS 500TM camera (GE Healthcare Life sciences).

From this original image we generated the image in the Fig 6C.

WB Arl8b – Fig 7C

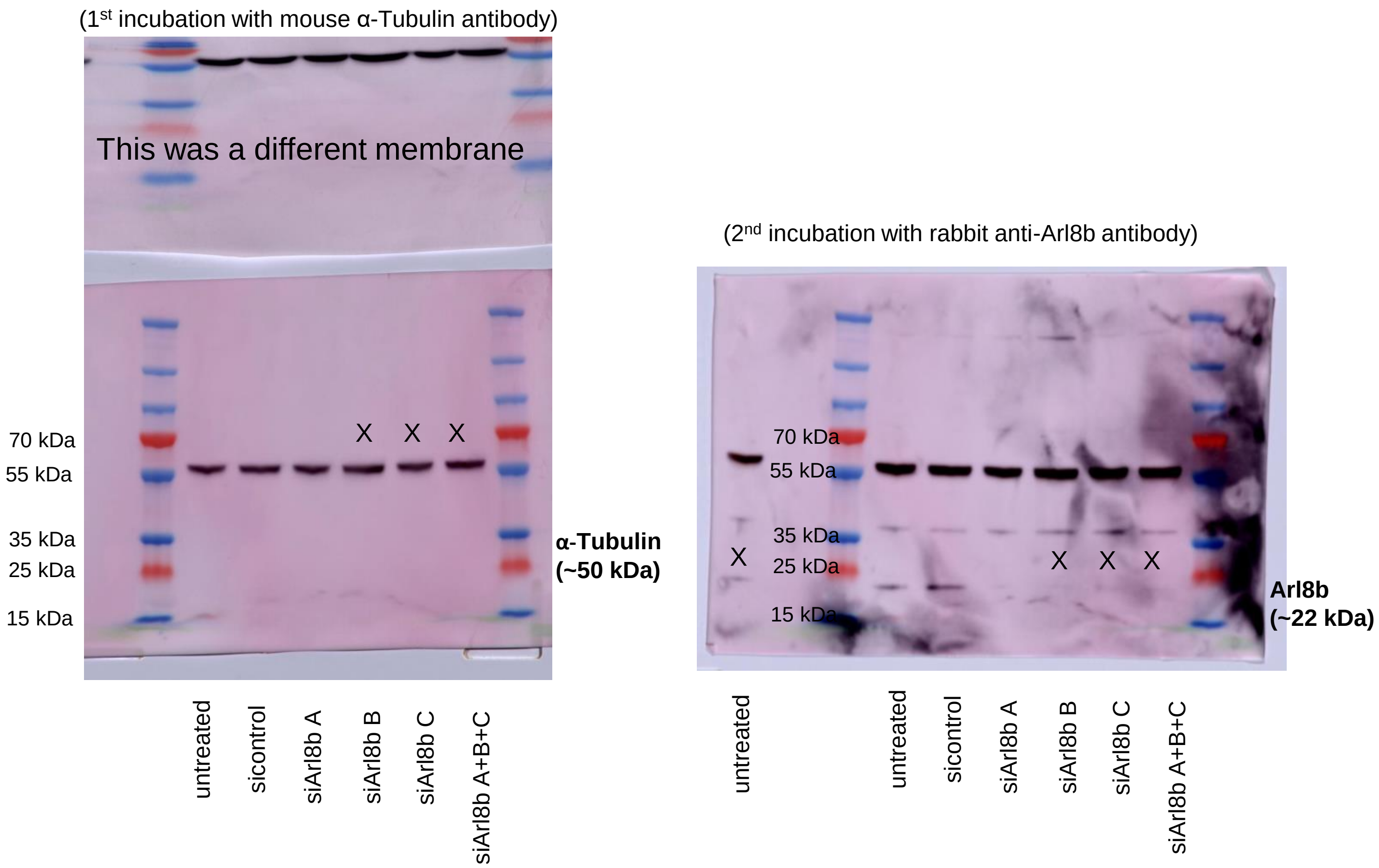

Cell lysates (30 µg/lane) of CAD cells untreated (untreated) or treated for 60 h with siRNA scramble (sicontrol)/siRNA Arl8b (siArl8b).

The specific protein bands were visualized using the ECL-immunoblotting chemiluminescence system (GE Healthcare Life sciences) and the ImageQuant LAS 500TM camera (GE Healthcare Life sciences).

From this original image we generated the image in the Fig 7C.
