# Supplementary material for: α-Synuclein fibrils subvert lysosome structure and function for the propagation of protein misfolding between cells through tunneling nanotubes
Source: PLoS Biol. 2021 Jul 20;19(7):e3001287. doi: 10.1371/journal.pbio.3001287 (PMC8291706; doi:10.1371/journal.pbio.3001287)
Supplement: Attachment — Submitted filename: Response to Reviewers.docx [file pbio.3001287.s012.docx]

REVIEWERS' COMMENTS:

Reviewer #1:

[identifies himself as Carmine Settembre]

The work entitled "α-synuclein fibrils affect lysosome structure, function and positioning to propagate misfolding through tunneling nanotubes" by Chiara Zurzolo's group describes in great details the functional relationship between α-synuclein and lysosomes. In particular they have investigated the consequences of α-synuclein accumulation on lysosome functions and how lysosomes participates to aggregate formation and spreading of α-synuclein. This is an important work, which might have implications for the understanding of basic principles behind the pathogenesis of α-synucleinopathies, such as Parkinson disease. The authors performed a detailed characterization of the described pathway through the extensive use of very high quality microscopy approaches. The data are crystal clear. The manuscript is very well written and easy to follow.
 
In my view the manuscript can be accepted in this current form. However, I would like to suggest two additional experiments that, in my opinion, might further improve the relevance and the mechanistic insights of this elegant study.

1) There is a known relationship between Parkinson and Gaucher disease, a lysosomal storage disorders caused by mutations in GBA. Notably, heterozygous Gaucher carriers have an increased risk to develop Parkinson. The observations that α-synuclein accumulation impairs lysosomal functions suggest a synergistic effect between a-syn and GBA haploinsufficiency. I think it would be interesting to study whether α-synuclein dependent lysosomal dysfunction and LMP is exacerbated in GBA-KO cells and whether these cells have enhanced spreading.

We thank the Reviewer for this comment. Indeed, α-syn-induced lysosomal dysfunction (e.g., LMP, failure in the activity of the lysosomal enzymes, etc.) shares a lot in common with Lysosomal Storage Disorders (LSDs). Moreover, the relationship between the defective activity of the lysosomal enzyme glucocerebrosidase (encoded by the GBA gene) and α-syn accumulation in Parkinson’s disease is an important area of study nowadays (Behl et al., Transl. Neurodegen., 2021). Actually, we have an ongoing project in the lab that focuses on LSDs, which also aims to investigate the link and the possible effects of α-syn fibrils in these diseases. Thus, even though we agree that it would be very interesting to study whether α-syn dependent lysosomal dysfunction and LMP is exacerbated in GBA-KO cells, and in line with the Editor’s suggestion, we prefer to save this suggestion for our ongoing project.

2) The induction of LMP has been recently shown promote nuclear translocation of TFEB (Nakamura et al. NCB 2020), which is known to influence lysosomal motility and exocytosis (Medina et al. DEV CELL 2015 and many others). Furthermore, TFEB overexpression promotes a-syn clearance (Decressac 2013 PNAS). Thus, I think it might be interesting to check whether the lysosomal loading of a-synuclein triggers TFEB nuclear translocation

and whether this (TFEB nuclear translocation) participates to lysosomal peripheral distribution upon a-synuclein loading.

We thank the Reviewer for raising this important point. Indeed, TFEB is known to be the master organizer of lysosomal biogenesis and autophagy and reported to be involved in the lysosomal distribution and clearance of the fibrils once activated (Medina et al., Dev Cell, 2011; Willett et al., Nat Comm, 2017; Zhang et al., J Biol Chem, 2017; Decressac PNAS, 2013; Kilpatrick et al., PLOS One, 2015). In light of these findings and following the Reviewer’s suggestion, to address whether α-syn fibrils trigger TFEB translocation to the nucleus, we transfected CAD cells with wild-type TFEB-GFP (TFEB-WT) and treated them with or without α-syn fibrils (18 h). We then quantified the presence of TFEB in the nucleus in these conditions. Interestingly, we found that α-syn fibrils induced TFEB translocation to the nucleus in CAD cells overexpressing TFEB-WT. We added this conclusion to page 18, lines 523-531, of the manuscript. The related data were presented in Fig 6A, and the Figure legend was updated accordingly (page 19, lines 551-554).

Concerning the second point raised by the Reviewer, we investigated whether TFEB had an effect on the peripheral distribution of lysosomes by using up- and down-regulation strategies. First, we overexpressed TFEB-WT-GFP and TFEB-mut-GFP (constitutively active form of TFEB that carries two mutations on S142/211A) in CAD cells and quantified the distribution of lysosomes compared to control CAD cells transfected with CMV-GFP. We detected more peripheral lysosomes both in TFEB-WT-GFP and TFEB-mut-GFP overexpressing CAD cells as compared to control cells expressing CMV-GFP. However, only TFEB-mut overexpressing cells were significantly different than the control condition suggesting that active form of TFEB promotes the peripheral distribution of lysosomes in CAD cells.

As a general observation, we have detected a tendency to having more peripheral lysosomes in all these conditions including control cells, suggesting that transfection itself might have an effect on lysosomal positioning, possibly because of the fact that it induces stress in the cells.

Next, to assess whether TFEB participates in lysosomal peripheral distribution upon α-syn loading, we downregulated TFEB in CAD cells by using specific siRNAs against TFEB (siTFEB). Following the evaluation of the siRNA efficiency by WB (around 100%), we analysed the distribution of lysosomes in control cells (treated with scrambled siRNA, sicontrol) and siTFEB cells in the presence or absence of α-syn fibrils. As expected, we observed that lysosomes containing α-syn in control condition were more peripheral, whereas in siTFEB cells these lysosomes were more perinuclear. These results indicate that the downregulation of endogenous TFEB impairs the redistribution of α-syn-containing lysosomes to the cell periphery, suggesting the involvement of TFEB in this process. These findings have been now added to pages 18-19, lines 532-549 of the manuscript. The related data were presented in Fig 6B-D, and the Figure legend was updated accordingly (page 19, lines 554-569). These results are also discussed on pages 33-34, lines 1018-1035.

Previous reports suggested that TFEB-regulated transcription induces lysosomal docking to the plasma membrane, thus promoting the peripheral distribution of lysosomes in HeLa cells (Medina et al., Dev. Cell., 2011). In contrast, TFEB and TFE3 have been reported to contribute to the perinuclear positioning of the lysosomes through transcriptional activation of TMEM55B in starvation conditions in HeLa cells (Willett et al. Nat. Comm., 2017). It has also been reported that overexpression of TFEB promotes perinuclear positioning of the lysosomes in mouse fibroblast cells defective for the lysosomal transporter cystinosin (Zhang et al., JBC., 2017). Our own data indicate that TFEB is involved in the peripheral distribution of the lysosomes containing α-syn fibrils; however, the mechanism for this event remains to be clarified. Given these controversies between published data, the role of TFEB on lysosomal positioning is still an open question and it would be very interesting to continue investigating its role in the context of α-syn and Parkinson’s Disease in general.

Reviewer #2:

The paper by Senol et al proposes that lysosomes play key roles in the transfer of pathogenic alpha-synuclein fibrils between neuronal cells. Through a combination of super-resolution imaging and electron microscopy, the authors provide evidence that lysosomal membrane permeabilization is key for the intracellular propagation of fibrils taken up from the culture medium. Moreover, they propose that lysosomes loaded with alpha-synuclein fibrils can move between cells via nanotubes connecting donor and acceptor cells. 

Overall, the manuscript proposes an intriguing model for alpha-synuclein propagation that is supported by high-quality experiments. 

Some points require additional clarification and/or experimental support, as detailed below.

1- Fig. 4 shows fibril-induced formation of a-Syn-GFP puncta, which are furthermore shown to localize near or within lysosomes. This is interpreted as evidence of seeding at lysosomes, however analysis was conducted at a single time point post-treatment (18h), thus it is still possible that a-Syn-GFP puncta may have formed elsewhere in the cytoplasm and subsequently migrated or be transported to lysosomes. A more detailed analysis with multiple time points should be carried out.

We thank the Reviewer for acknowledging the quality of our data and for raising this important point. To determine when the seeding begins in CAD cells, we designed an experiment where we could follow the seeding process in living cells. Thus, we first transfected CAD cells with a fluorescent reporter of soluble α-syn (α-syn-GFP) and after the addition of Alexa 568-tagged α-syn fibrils, we immediately started monitoring the seeding event by detecting the formation of green puncta (newly formed α-syn-GFP aggregates) by spinning disk microscopy in comparison to control cells (to check for spontaneous formation of α-syn-GFP puncta in the absence of α-syn fibrils). Seeding started to become apparent after about 2h from the addition of the α-syn fibrils and increased with time as shown in the new S1 Movie and S5A Fig now added to the manuscript. We did not detect any spontaneous formation of α-syn-GFP puncta in control cells (new S2 Movie and S5B Fig now added to the manuscript). Next, we performed super resolution microscopy to investigate whether or not these nascent aggregates were associated with lysosomes at different time points (1h, 2h, and 4h). At all of these time points, we found that newly formed aggregates co-localized with α-syn fibrils and LAMP1 (lysosomal marker). Therefore, these data strongly indicate that seeding occurs at lysosomes and strengthen our hypothesis that lysosomes function as hubs for the seeding of newly formed α-syn aggregates. We have now added these results to our manuscript (see page 13, lines 357-372), updated Fig 4, created a new Supplementary Fig (S5 Fig), and added new Supplementary Movies (Movies S1 and S2). Figure legends (page 14, lines 392-402; page 64, lines 1992-1996; page 67, lines 2064-2072) and the Materials and Methods section (pages 44-45, lines 1369-1376) have been updated accordingly.

2- Given the key role of lysosomes as carriers of a-Syn fibrils, does increasing the number of lysosomes (i.e. via overexpression of the master regulator of lysosomal biogenesis, TFEB transcription factor) increase the efficiency of transfer and/or seeding?

We thank the Reviewer for this interesting comment, which is also in line with the suggestions of Reviewer #1.

However, we found that overexpression of WT TFEB and its active mutant do not increase the number of lysosomes in CAD cells (Graph A, below). Nonetheless, by following the suggestion of Reviewer #1, we found that translocation of TFEB induced the peripheral distribution of lysosomes containing α-syn fibrils (see reply point 2, and new Fig 6). Thus, next we investigated whether TFEB overexpression had any effect on the transfer of α-syn-fibrils and on seeding as suggested by the Reviewer #2. To this aim, we performed a co-culture experiment where TFEB-WT-GFP-, TFEB-mut-GFP- and CMV-GFP (control)-transfected cells loaded with α-syn fibrils were used as donors and H2B-mCherry transfected cells as acceptors. We then quantified the efficiency of α-syn transfer from donor to acceptor cells in these co-cultures. By analyzing the data collected from three independent experiments, we did not detect any significant difference in the transfer of α-syn between these co-culture conditions, suggesting that overexpression of TFEB (both WT and constitutively active mutant) has no effect on the transfer efficiency of α-syn fibrils (Graph B, below).

B

A

We then addressed whether overexpression of TFEB had an effect on seeding and thus performed an experiment where CAD cells were co-transfected with α-syn-GFP and TFEB-mCherry (WT or constitutively active mutant) and loaded with non-tagged α-syn fibrils or not. We then quantified the percentage of cells that formed new aggregates in control and α-syn-treated cells. Data collected from three independent experiments showed that seeding occurred only in the conditions where cells were treated with α-syn fibrils and this event was not affected by overexpression of TFEB-WT or TFEB-mut, suggesting that TFEB doesn’t play a role in seeding (Graph below).

As these are negative data, we prefer not to include them in the manuscript. However, if this referee believes that they are relevant we could add them to complete the data presented in Fig 6, as Supplementary Information.

3- a-Syn is shown to cause LMP, which was previously proposed to trigger an autophagy-based repair pathway known as lysophagy (PMID: 23921551). Does a-Syn fibril uptake result in lysophagy (defined as engulfment of Ubiquitin-positive LAMP1-positive lysosomes inside LC3 structures)? Also, does autophagy promote or antagonize a-Syn induced LMP and the transfer of a-Syn-loaded lysosomes between donor and acceptor cells? 

We thank the Reviewer for these questions. To assess whether α-syn fibrils trigger lysophagy, we performed the immunofluorescence microscopy analysis suggested by this Reviewer in CAD cells treated or not with Alexa 568-tagged α-syn fibrils. As a positive control, we used LLOMe, a well-known inducer of LMP. Cells were then labeled for Ubiquitin, LC3 and LAMP1, and colocalization of these markers was analyzed. Data collected from three independent experiments revealed that in control cells we could only detect a few LC3 and ubiquitin puncta; however, we were not able to detect any colocalization between these puncta and lysosomes. On the other hand, in CAD cells treated with LLOMe, we could detect nice colocalization between LC3, ubiquitin and LAMP1, as expected. However, in α-syn fibrils-treated cells we could detect only one lysosome in three experiments (average 60 cells per experiment analysed) that was colocalized with LC3 and Ubiquitin; thus, lysophagy was an extremely rare event. Taken together these data suggest that α-syn fibrils do not induce lysophagy. We now present these data as Supplementary Fig (S4 Fig) in the revised version of the manuscript (page 11, lines 297-308). Figure legend (page 64, lines 1986-1990) and Materials and Methods section (page 40, lines 1212-1223) have been updated accordingly. In addition, we discuss these findings in the Discussion section (page 32, lines 976-982).

To answer the question whether autophagy promotes or antagonizes α-syn-induced LMP, we designed an experiment where we transfected CAD cells with Gal3-GFP, treated these cells with α-syn fibrils (or not), and in addition treated them with bafilomycin (100 nM, 4 hours) to block autophagic degradation as described before (Chastagner et al., EMBO Mol.Med., 2020). Next, we quantified the Gal3-GFP puncta formation upon LMP in all of these conditions. We found that, in the presence of α-syn fibrils, bafilomycin treatment significantly increased the percentage of cells under LMP compared to control cells treated with bafilomycin or DMSO, indicating that blocking autophagy increases LMP (see the Graph and the Table below). Overall, these findings suggest that autophagy antagonizes α-syn induced LMP. Our results are in line with previous reports where calpain-mediated LMP was shown to underlie lysosomal impairment and decreased autophagy (Rodriguez-Muela et al., Cell Death & Differentiation, 2015), and impaired autophagic flux was shown to contribute to the occurrence of LMP (Cui et al., Front. Cell Dev. Biol., 2020).

It is important to note that the percentage of LMP in control cells treated only with DMSO (30%) was much higher than the control cells without any treatment (10%; Fig 3); therefore, even though we detected a tendency to increased LMP in α-syn treated cells, the results were not significantly different. This suggests that DMSO treatment itself can increase LMP in control cells possibly due to stress. We did not observe any significant difference in the average number of Gal3-GFP puncta detected between these conditions (see the Graph below).

Also in this case, we do not believe it will be useful to add these data to the paper. However, we prepared a complete figure above for this Reviewer, and if he/she thinks that it will be good to add these data, we could include them as a Supplementary Figure.

Finally, to answer the question whether autophagy promotes or antagonizes α-syn-induced transfer of α-syn-loaded lysosomes between donor and acceptor cells, we performed co-culture experiments where we used as donors CAD cells overexpressing ATG5-GFP that has been reported to induce autophagy (Levia-Rodriguez et al., Cell Death & Disease, 2018; Pyo et al., Nat. Comm., 2013; Zheng et al., Cell. Commun. Signal., 2019). We loaded CAD cells (control co-culture) or CAD ATG5-GFP cells with α-syn fibrils and co-cultured them with HeLa cells transfected with H2B-mCherry. We then stained this co-culture with anti-LAMP1 mouse antibody, to assess specifically the transfer of donors’ lysosomes to acceptor cells. By quantifying the percentage of acceptor HeLa cells that received α-syn fibrils or lysosomes originated from donor cells, we did not observe any significant difference between co-cultures having ATG5-GFP overexpressing donors and control donors loaded only with α-syn fibrils (Graphs A and C respectively, below). In addition, the average number of α-syn puncta or lysosomes detected in these acceptors remained unchanged (Graphs B and D respectively, below). Finally, we did not detect any difference in the percentage of lysosomes containing α-syn fibrils in these co-cultures (Graph E, below). Therefore, we concluded that increasing autophagy, via overexpression of ATG5, does not have an effect on the transfer of α-syn-containing lysosomes from donor to acceptor cells. These data are illustrated below for the referees, but we believe they should not be included in the paper.

A

E

B

D

C

Overall, our data on autophagy indicated that it can indeed antagonize α-syn-induced LMP but it has no effect on the transfer of α-syn fibrils or lysosomes. We believe that these questions should be further addressed by using different methods and investigated in more detail to be conclusive. We have added this comment in the Discussion of the manuscript (page 32, lines 978-982).

Reviewer #3:
 
This revised comprehensive study employed an array of imaging techniques as well as other assays to probe the role of lysosomes in the spreading a-syn pathology from cell to cell. This is an area of great interest to neuroscientists who study neurodegeneration. Some of their findings are that a-syn fibrils affect the morphology, function and distribution of lysosomes within CAD (mouse catecholaminergic) cells, and that lysosomes containing a-syn fibrils can be transferred from a donor cell to a recipient. Moreover, because a-syn permeabilizes the lysosomal membrane, once a-syn fibril+/lysosomes are transferred into a naïve recipient cell, the fibrils apparently can escape the compartment and catalyze fibrillization of endogenous, non-toxic a-syn. The lysosomes are transferred from cell to cell via tunneling nanotubes. Points to address are given below. 


Strengths

An array of imaging techniques show a-syn fibrils in or on lysosomes
Functionality of lysosomes were measured by a pH sensitive marker and cathepsin activity
Morphology of lysosomes evaluated by TEM and other imaging techniques
Movement of lysosomes between donor and recipient cells tracked by following different labels.
Attention paid to statistical evaluation of differences in readouts

Weaknesses / or points needing clarification

1) p. 12, lines 417-425:

"Since our results indicate that α-syn fibrils affect lysosomal pH and function (Fig 2 B and D),
to understand whether there is a correlation between lysosome distribution and their
functionality, we next evaluated the distribution of lysosomes containing α-syn fibrils positive
for LysoTracker DR and Magic Red CathB. We found that α-syn fibril-containing LysoTracker
DR and Magic Red CathB positive lysosomes were more perinuclear (54 ± 2% and 56 ± 1%,
respectively; Fig 5 C), suggesting that peripheral lysosomes containing α-syn fibrils are less
functional.  

These results show that α-syn fibrils affect lysosomes distribution towards the cell periphery.
Furthermore, this pool of peripheral lysosomes is less acidic and has less degradative capacity."

Looking at Fig. 5C, it is hard to understand the  sentence, "We found that α-syn fibril-containing LysoTracker DR and Magic Red CathB positive lysosomes were more perinuclear (54 ± 2% and 56 ± 1%, respectively; Fig 5 C), suggesting that peripheral lysosomes containing α-syn fibrils are less functional."  

In the plot, it looks like the perinuclear and peripheral lysosomes have the same functionality.

We thank the Reviewer for bringing up this point, which we realize was not clearly explained in the first version of the manuscript. Indeed, we have actually quantified only the “functional” lysosomes, that is, the ones that are positive for Lysotracker and Magic Red and compared their perinuclear and peripheral distribution. To clarify this point, we modified the text as follows: “Since our results indicate that α-syn fibrils affect lysosomal pH and function (Fig 2B-D), to understand whether there is a correlation between lysosome distribution and function, we next evaluated the distribution of lysosomes containing α-syn fibrils and quantified the perinuclear and peripheral distribution of only functional lysosomes positive for LysoTracker DR and Magic Red CathB. We found that α-syn fibril-containing LysoTracker DR positive and Magic Red CathB positive lysosomes were more perinuclear (54 ± 2% and 56 ± 1%, respectively; Fig 5C), suggesting that peripheral lysosomes containing α-syn fibrils are less functional.” (page 15, lines 422-428).

In addition, we have updated the presentation of the graph in Fig 5C and added the statistical comparison between perinuclear and peripheral distribution of both LysoTracker DR positive and Magic Red CathB positive puncta (instead of comparing only the perinuclear and peripheral distribution of LysoTracker DR and Magic Red CathB among themselves).

2) Discussion, p. 30, lines 972-975: 

"However, as we and others demonstrated that cells form more TNTs under stress such as when
they are treated with H202 to increase oxidative stress (107, 108). In line with these findings,
and more specifically, we have previously reported that CAD cells bearing α-syn fibrils exhibit
sustained increase in ROS levels (18). Therefore, α-syn fibrils can indeed trigger stress in the
cells in order to induce TNT formation."

Cancer cells redistribute lysosomes upon stress from the perinuclear region to the periphery (Steffan J Cell Sci 2010, 123: 1151). It seems the same thing is happening here. HeLa cells are cancer cells. Are CAD cells cancer cells ? It is not clear to this reviewer what type of cell they are. It says in the methods that they were used undifferentiated, perhaps this implies that these cells are like SH-SY5Y cells, which are cancer cells.

To clarify this point, we added detailed information about CAD cells and explained that indeed they are cancer cells but can exist in both differentiated and undifferentiated state depending on the absence or presence of serum in their medium, respectively. In support of this explanation, we cited the original paper where CAD cells are characterized (Qi et al., J. Neurosci., 1997; see Materials and Methods section page 37, lines 1116-1123).

Questions:

a) Does lysosome trafficking occur between differentiated donor and differentiated acceptor cells?

We have previously reported that α-syn fibrils can be efficiently taken up by primary neurons, transferred from donor to acceptor neurons in a co-culture system, and localized to lysosomes in both donor and acceptor cells (Abounit et al. EMBO J., 2016, Loria et al., Acta Neuropathol., 2017). Thus, we can conclude that lysosome trafficking occurs between differentiated donor and acceptor cells.

b) If lysosome redistribution occurs in cancer cells due to stress, then it is quite likely that lysosome redistribution occurs in the CAD cells due to the stress (increase in ROS?), as stated above. It seems reasonable to determine whether an antioxidant like NAC or TEMPO would inhibit lysosome redistribution in CAD cells treated with a-syn fibrils. Pharmacologically inhibiting lysosomal redistribution to the periphery of fibril-treated donor cells should decrease transfer to naive recipient cells.

We thank the Reviewer for this suggestion. We have previously reported that increased ROS levels can be detected upon α-syn fibril uptake in neuron-like CAD cells (Abounit et al., EMBO J., 2016) and in the current study we demonstrated that α-syn treatment induces lysosomal repositioning toward the cell periphery. Thus, before performing the experiment suggested, we evaluated whether increased ROS levels would affect lysosomal distribution per se in CAD cells. To this end, we treated CAD cells with H_2_O_2_ (1 mM, 4 h) and we evaluated the lysosomal distribution in H_2_O_2_-treated cells versus untreated control cells. We did not observe any difference in the perinuclear and peripheral distribution of lysosomes in these conditions (see the Graph below). Therefore, we have concluded that it is α-syn fibrils and not ROS levels that affect lysosomal distribution in CAD cells. For this reason, we did not perform further experiments to investigate this point that at the moment is outside the scope of the paper.

c) In the discussion, it would be helpful if the authors talked about the limitations of their model, i.e.,

i) that cancer cells are used.

ii) if differentiated CAD cells are not optimal to study TNTs (methods section), isn't the probability low that Lewy body pathology spreads from cell to cell via TNT-guided lysosomes in vivo? You do not have to say it the way it is phrased in this sentence. It's just that you should provide the Reader with the limitations of your work.

We thank the Reviewer for raising these points, as they are indeed important. We therefore added the following paragraphs in the Discussion of the manuscript (pages 35-36, lines 1091-1100). We have previously reported that α-syn fibrils can be efficiently transferred between neurons, primary astrocytes, neurons and astrocytes, astrocytes and organotypic hippocampal slices and between iPSCs and they were associated to lysosomes in these co-culture systems (Abounit et al., 2016; Loria et al., Acta Neuropathol., 2017; Grudina et al., Neurobiol. Dis., 2019); however, we have used here undifferentiated neuronal CAD cells line, for their easy manipulation. Although our findings contribute to a better understanding of the mechanism of development and progression of α-syn pathology opening new avenues for possible therapeutic approaches against synucleinopathies, it will be necessary in the future to investigate the transfer of α-syn containing lysosomes in differentiated primary neurons and/or in ex-vivo/in-vivo systems to further validate our data and overcome the limitation of this study.

3). Bar graphs: Fig. 1A;  Fig. 2A-D;  Fig. 3 B, C;  Fig. 4 B,F;  Fig. 5 B-E, G,H; Fig. 6 A,C,E,F; and so on.

It would greatly improve the manuscript if the authors showed all data points for each condition rather than just bars with the attached error bar.

We thank to the Reviewer for this suggestion. All graphs which had been presented as bar graphs are now replaced by “all data points” as suggested. Specifically, graphs in Figs 1A, 2A (right graph), 2B, 2C, 2D, 3B (left graph), 3C, 4B (left graph), 4F, 5B, 5C, 5D, 5E, 5G (left graph), 5H, 7A (left graph), 7C, 7E (left graph), 7F, 8B (upper graph), 9B (left graph), 9G, 10B (left graph), 10D (left graph), 10G (both right left graphs), 10I (left graph); and Supplementary Figs S6B (left graph), S6D (left graph), S7B (left graph), S7E (left graph).

In Fig. 9 D, G and E (left-hand plots), it would help the Reader if the y-axis ranges were ~40 rather than 100.

Related to the changes mentioned above in the graphs, once all data points were used for the graph presentations, the distribution of the data reached above 40, thus we had to keep the y-axis at 100.

**Detailed list of changes in the manuscript:**

**Figures:**

**Fig 1:** in panel A the bar graph has been replaced with the all data points graph.

**Fig 2:** the bar graphs in panels A (right graph), B, C, and D have been replaced with all data points graphs.

**Fig 3:** the bar graphs in panels B (left graph) and C have been replaced with all data points graphs.

**Fig 4:** the bar graphs in panels B (left graph) and F have been replaced with all data points graphs.

Super resolution microscopy images demonstrating α-syn fibrils puncta and α-syn-GFP+ aggregate colocalizing with LAMP1 on the lysosomal membrane in CAD cells treated with α-syn fibrils for 1 h, 2 h, and 4 h have been added in panel G, H, and I respectively.

**Fig 5:** the bar graphs in panels B, C, D, E, G (left graph), and H have been replaced with the all data points graphs.

**Fig 6:** a new figure has been created to demonstrate the role of TFEB in lysosome redistribution upon α-syn loading.

Panel A: graph representing the percentage of cells overexpressing TFEB-WT-GFP having nuclear TFEB in control CAD cells and α-syn fibril-treated CAD cells for 18 h.

Panel B: graph representing the percentage of LAMP1+ lysosome distribution in perinuclear and peripheral regions of the control cells overexpressing CMV-GFP, TFEB-WT-GFP, or TFEB-mut-GFP.

Panel C: Western blot prepared from cell extracts collected from control, scrambled siRNA (sicontrol), and siTFEB pretreated CAD cells for 60 h, showing TFEB and GAPDH (loading control) expression.

Panel D: graph representing the distribution of lysosomes containing α-syn fibrils in perinuclear and peripheral region of α-syn fibril-treated sicontrol and siTFEB cells.

**Fig 7:** original Fig 6 of the manuscript has been changed to Fig 7 and the bar graphs in panels B (left graph), C, E (left graph), and F have been replaced with the all data points graphs.

**Fig 8:** original Fig 7 of the manuscript has been changed to Fig 8 and the bar graph in panel B (upper graph) has been replaced with the all data points graph.

**Fig 9:** original Fig 8 of the manuscript has been changed to Fig 9 and the bar graphs in panels B (left graph) and G have been replaced with all data points graphs.

**Fig 10:** original Fig 9 of the manuscript has been changed to Fig 10 and the bar graphs in panels B (left graph), D (left graph), G (left graph), and I (left graph) have been replaced with the all data points graphs.

**Fig 11:** original Fig 10 of the manuscript has been changed to Fig 11 and the working model has been updated according to the new findings concerning the role of TFEB in lysosome redistribution upon α-syn loading.

**Supplementary Fig 3:** in panel A the schematic has been updated including the transfection with TFEB-WT-GFP (number 3).

**Supplementary Fig 4:** a new figure has been created to demonstrate that α-syn fibrils do not induce lysophagy.

**Supplementary Fig 5:** a new figure has been created to demonstrate that the seeding began to become apparent after about 2 h from the addition of the α-syn fibrils and increased with time (timeframes of the Movies S1 and S2).

Panel A: timeframes at 0/1/2/3/4 hours of the S1 Movie of CAD cells overexpressing soluble α-syn-GFP monitored after the administration of α-syn fibrils.

Panel B: timeframes at 0/1/2/3/4 hours of the S2 Movie of CAD cells overexpressing soluble α-syn-GFP.

**Supplementary Fig 6:** original Supplementary Fig 4 of the manuscript has been changed to Supplementary Fig 6 and the bar graphs in panels B (left graph) and D (left graph) have been replaced with the all data points graphs.

**Supplementary Fig 7:** original Supplementary Fig 5 of the manuscript has been changed to Supplementary Fig 7 and the bar graphs in panels B (left graph), E (left graph), and I have been replaced with the all data points graphs.

**Supplementary Movie 1**: a new movie has been provided to demonstrate that the seeding began to become apparent after about 2 h from the addition of the α-syn fibrils and increased with time in CAD cells expressing soluble α-syn-GFP.

**Supplementary Movie 2**: a new movie has been provided showing that the seeding did not occur in CAD cells expressing soluble α-syn-GFP not treated with α-syn fibrils.

**Text:**

The entire text has been proofread for minor corrections and typos. The revised/new text has been highlighted throughout the paper, with the major edits summarized below.

**Introduction:**

Page 4: we have updated the text according to the new results on the role of TFEB on lysosome redistribution upon α-syn fibrils loading.

**Results:**

Page 11 (chapter entitled: “Lysosomes containing α-syn fibrils undergo Lysosomal Membrane Permeabilization (LMP)”): new results showing that lysosomes containing α-syn fibrils are not targeted to lysophagy have been added.

Page 13 (chapter entitled: “α-Syn fibrils seed de novo formation of aggregates at the lysosomes”): new results showing that the seeding of newly formed α-syn-GFP aggregates in CAD cells expressing soluble α-syn-GFP began to become apparent after about 2 h from the addition of the α-syn fibrils and increased with time have been added.

Page 15 (chapter entitled: “α-Syn loaded lysosomes localize at the cell periphery and are less functional”): the sentence describing the evaluation of the distribution of only the functional lysosomes (i.e., positive for LysoTracker DR and Magic Red CathB) has been modified in order to make it clearer.

Pages 18-19: a new chapter entitled “TFEB as a player in lysosomes’ redistribution upon α-syn loading” has been added to describe the new results concerning the role of TFEB in the lysosomes redistribution upon α-syn loading.

**Discussion:** the discussion section has been updated considering the new results obtained.

**Material and methods:** The chapter entitled “Cell culture, transfection, siRNA treatment” has been updated by adding detailed information about CAD cells and the siRNA treatment to downregulate TFEB.

Additional chapters have been added to describe the methodology used in new experiments: immunocytochemistry for lysophagy (page 40), Live imaging microscopy (pages 44-45).

**Figure legends:** Figure legends have been rewritten and updated according to the novel results.
